# Endogenous Histones Function as Alarmins in Sterile Inflammatory Liver Injury Through Toll-like Receptor 9 in Mice

**DOI:** 10.1002/hep.24501

**Published:** 2011-08-25

**Authors:** Hai Huang, John Evankovich, Wei Yan, Gary Nace, Lemeng Zhang, Mark Ross, Xinghua Liao, Timothy Billiar, Jun Xu, Charles T Esmon, Allan Tsung

**Affiliations:** 1 Department of Surgery, University of Pittsburgh Medical CenterPittsburgh, PA; 2 Department of Cell Biology, University of Pittsburgh Medical CenterPittsburgh, PA; 3 Department of Cardiovascular Biology Research Program, Oklahoma Medical FoundationOklahoma City, OK; 4 Howard Hughes Medical Institute, Departments of Pathology and Biochemistry and Molecular Biology, University of Oklahoma Health Sciences CenterOklahoma City, OK

## Abstract

Sterile inflammatory insults are known to activate innate immunity and propagate organ damage through the recognition of extracellular damage-associated molecular pattern (DAMP) molecules. Although DAMPs such as endogenous DNA and nuclear high-mobility group box 1 have been shown to be critical in sterile inflammation, the role of nuclear histone proteins has not yet been investigated. We report that endogenous histones function as DAMPs after ischemic injury through the pattern recognition receptor Toll-like receptor (TLR) 9 to initiate inflammation. Using an *in vivo* model of hepatic ischemia/reperfusion (I/R) injury, we show that levels of circulating histones are significantly higher after I/R, and that histone neutralization significantly protects against injury. Injection of exogenous histones exacerbates I/R injury through cytotoxic effects mediated by TLR9 and MyD88. In addition, histone administration increases TLR9 activation, whereas neither TLR9 nor MyD88 mutant mice respond to exogenous histones. Furthermore, we demonstrate *in vitro* that extracellular histones enhance DNA-mediated TLR9 activation in immune cells through a direct interaction. *Conclusion:* These novel findings reveal that histones represent a new class of DAMP molecules and serve as a crucial link between initial damage and activation of innate immunity during sterile inflammation. (Hepatology 2011; 54:999–1008)

Although foreign pathogens and their products, pathogen-associated molecular pattern (PAMP) molecules, have been shown to activate the innate immune system, the mechanisms by which damaged tissues notify the immune system remain to be fully elucidated. The recognition of a group of endogenous damage-associated molecular pattern (DAMP) molecules that serve a similar function to PAMPs has provided a framework for understanding the overlap between the inflammatory responses activated by pathogens and injury. It appears that certain pattern recognition receptors (PRRs), such as the family of Toll-like receptors (TLRs), serve as a common pathway for immune recognition of microbial invasion and tissue injury. By recognizing either PAMP or DAMP molecules, PRRs alert the host to tissue damage by activating the innate immune system. Initially, this process is manifested by the production of inflammatory mediators that allow the host to respond appropriately to infectious or noninfectious insults. When excessive, this inflammatory response can contribute to severe organ damage and dysfunction.

Several DAMPs have been identified, including heat shock proteins, S100 proteins, hyaluronan, heparin sulfate, RNA, DNA, and high-mobility group box 1 (HMGB1).^1^ Endogenous DAMPs are elevated and contribute to poor outcomes in several inflammatory models, both infectious and noninfectious, including sepsis,^2^ acute lung injury,^3^ pancreatitis,^4^ burns,^5^ and trauma.^6^, ^7^ These molecules are recognized by PRRs on various cell types, and in turn, drive the inflammatory response. We previously identified the endogenous DAMP HMGB and the PRR TLR4 to be major mediators of organ damage in hepatic ischemia/reperfusion (I/R).^8^ Recently, DNA and TLR9 have also been shown to play critical roles.^9^ Although studies by our group and by others have investigated the function of circulating HMGB1 and DNA in I/R injury, the involvement of histone proteins, which are closely associated with both HMGB1 and DNA in the nucleus, has not yet been examined. Traditionally, histones have been examined in the context of regulating nuclear architecture; however, recent reports have also examined the role of extracellular histones.^10^–^12^ Most importantly, Xu et al.^13^ identified extracellular histones as major mediators of death in sepsis.

I/R injury consists of a series of pathophysiological events that involve deprivation of blood and oxygen followed by their restoration. Liver I/R injury occurs unavoidably after elective liver resection, organ transplantation, trauma, and hypovolemic shock.^14^ Subsequent organ damage occurs as a result of both direct cellular damage, including hepatocyte necrosis and apoptosis, as well as delayed organ dysfunction from activation of the innate immune system and propagation of the inflammatory response.^15^–^18^ Although much is known, the roles of endogenous DAMPs and PRRs have not been fully delineated.

The aim of this study was to determine the initial mechanisms by which ischemic tissues alert the innate immune system in sterile tissue damage. We used I/R as a model of acute, noninfectious tissue injury to the liver. We demonstrate that extracellular histones are released from liver parenchymal cells *in vitro* and *in vivo*. Although exogenous administration of histones exacerbates I/R injury, neutralizing antibodies prevent hepatocellular damage and suppress the activation of the inflammatory cascade. These effects are mediated through TLR9 and MyD88. Furthermore, in addition to directly activating TLR9, extracellular histones also enhance nucleic acid–mediated inflammation through TLR9.

## Materials and Methods

### Materials

Antibodies to histone H3 (LG2-1) and histone H4 (BWA-3) were provided by Jun Xu.^13^ Immunoglobulin G (IgG) (item I5006), calf thymus histones (H9250), and Dnase I (D5319) were obtained from Sigma-Aldrich. TLR9 agonist CpG (ODN1826), antagonist CpG (ODN2088), or control CpG were obtained from Invitrogen. A commercial kit based on histone release was purchased from Roche.

### Animals

Male wild-type (C57BL/6) mice (8-12 weeks old) were purchased from Jackson ImmunoResearch Laboratories. TLR9^CpG/CpG^ mutant, MyD88^−/−^ and MyD88^+/+^ mice were provided by Timothy Billiar (University of Pittsburgh Medical Center, Pittsburgh, PA). Animal protocols were approved by the Animal Care and Use Committee of the University of Pittsburgh, and the experiments were performed in adherence to National Institutes of Health guidelines for the use of laboratory animals.

### Liver I/R

A nonlethal model of segmental (70%) hepatic warm ischemia and reperfusion was used as described.^19^

### Experimental Design

Mice received anti-histone H3 or H4 antibodies (20 mg/kg),^13^ or control IgG (Sigma-Aldrich) intravenously 30 minutes before ischemia. Histones (5 mg/kg and 25 mg/kg), CpG, ODN2088 (100 μg per mouse), or phosphate-buffered saline (PBS) were injected intraperitoneally immediately after ischemia. Sham animals underwent anesthesia, laparotomy, and exposure of the portal triad without hepatic ischemia. Animals were sacrificed at predetermined time points (1-6 hours).

### Isolation and Culture of Hepatocytes and Nonparenchymal Cells

Hepatocytes and nonparenchymal cells (NPCs) were isolated from normal wild-type (C57BL/6) mice as described.^8^, ^20^ Hepatocytes (3 × 10^6^) and NPCs (50 × 10^6^) were plated as described.^8^, ^20^

### *In Vitro* Coculture Assays

Wild-type hepatocytes were rendered necrotic by incubation at 60°C for 60 minutes as described.^9^ Supernatants from necrotic hepatocytes were harvested after a 12-hour incubation period at 37°C and were used as conditioned media in subsequent coculture assays.

### Liver Damage Assessment

Serum alanine aminotransferase (sALT) levels were measured using the DRI-CHEM 4000 Chemistry Analyzer System (Heska).

### Sodium Dodecyl Sulfate–Polyacrylamide Gel Electrophoresis and Western Blotting

Western blot analysis for histone H3 and H4 (Cell Signaling Technology), functional TLR9 (1:1,000; eBioscience), extracellular signal-regulated kinase, p38, c-Jun N-terminal kinase, and nuclear factor κB (NF-κB) p65 (1:1,000; Cell Signaling Technology) were performed as described.^21^, ^22^

### SYBR Green Real-Time Reverse-Transcription Polymerase Chain Reaction

Total RNA was extracted from the liver using the RNeasy Mini Kit (Qiagen). Messenger RNA (mRNA) for tumor necrosis factor α (TNF-α), interleukin-6 (IL-6), and β-actin was quantified in duplicate by SYBR Green (reverse-transcription polymerase chain reaction [PCR]). PCR reaction mixture was prepared using SYBR Green PCR Master Mix (PE Applied Biosystems) using described primers.^21^

### Immunofluorescent Staining

Cells were incubated with the specific primary antibodies for histone H3 and H4 (1:500; Abcam) in 1% bovine serum albumin for 1 hour, washed 4 times, and incubated with secondary antibody (1:500; AlexaFluor 488 goat anti-rabbit, Invitrogen). F-actin was stained with rhodamine phalloidin (Invitrogen). Cells were mounted with Vecta-Shield Mounting media with DAPI nuclear stain. Slides were viewed with Olympus Provis and Leica TSL-SL immunofluorescent microscopes.

### Coimmunoprecipitation

Immunoprecipitation was performed with 1 μg of antibodies against histone H4 in 300 μg whole lysate protein or 40 μL of serum diluted in immunoprecipitation buffer [50 mM 4-(2-hydroxyethyl)-1-piperazine ethanesulfonic acid, 0.5% Nonidet P-40, 150 mM NaCl, 10% glycerol, 1 mM ethylene diamine tetraacetic acid]. Normal rabbit IgG was used as a negative control. Precleared lysates were incubated with anti-TLR9 overnight, and then incubated for 2 hours with protein A/G-agarose. Samples were washed four times with PBS and subjected to western blot analysis.

### Statistical Analysis

Results are expressed as the mean ± SE. Statistical analysis was performed using the Student *t* test or one-way analysis of variance. All statistical analyses were performed using Sigma Stat version 3.5 (Systat Software, Inc.). Graphs were generated using Sigma Plot version 10 (Systat Software, Inc.). *P* < 0.05 was considered statistically significant.

## Results

### Neutralizing Extracellular Histones Is Protective in Hepatic I/R Injury

**Fig. 1:**
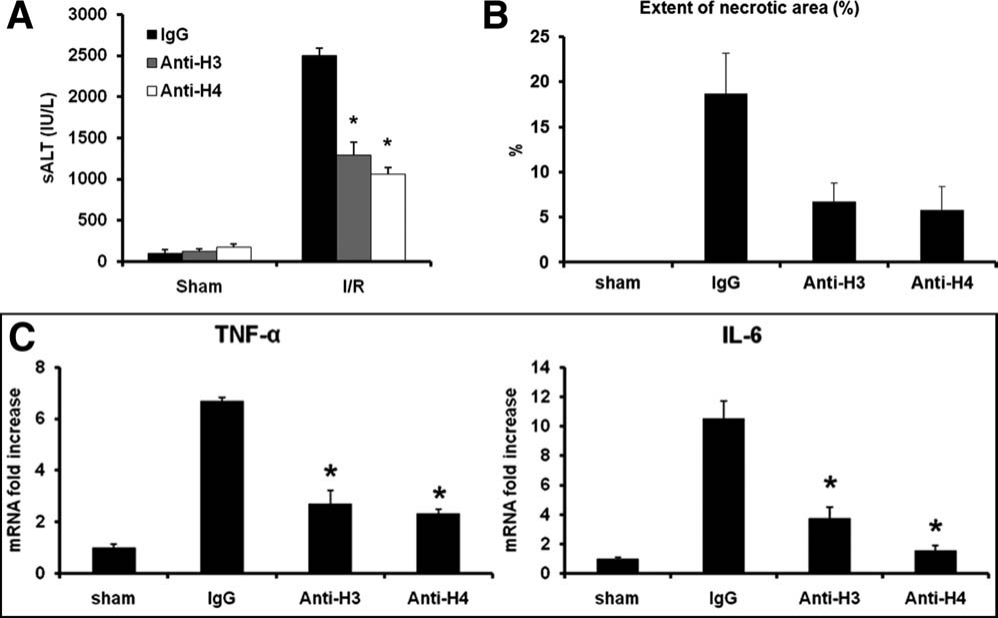
Pretreatment with neutralizing antibodies to histones protects against liver I/R injury. (A) Sham or I/R-treated mice were given anti-histone H3 or anti-histone H4 antibody (20 mg/kg body weight) or control antibody intravenously 30 minutes before ischemia. Data represent the mean ± SE (n = 6 mice per group). *P < 0.05. (B) Quantification of necrotic hepatocytes in hematoxylin and eosin–stained liver sections from control and anti-histone antibody-treated animals 6 hours after reperfusion. The graph is representative of liver sections from six mice per group. (C) Hepatic TNF-α and IL-6 mRNA expression after 6 hours of I/R. Results are expressed as relative increase of mRNA expression compared with sham-treated animals. Data represent the mean ± SE (n = 6 mice per group). *P < 0.05.

Nuclear histone proteins associate closely with DAMP molecules HMGB1 and DNA, both of which are known to impact I/R injury. Thus, to determine whether extracellular histones contribute to hepatic organ damage after hepatic I/R, neutralizing antibodies to histone H3 or H4 were administered to mice before I/R. Anti-H3 and anti-H4 antibodies had no effect on sham-treated animals but conferred significant protection after I/R (Fig. 1A,B). IgG-treated animals exhibited 18.7 ± 4.5% necrotic hepatocytes compared with 6.7 ± 2.1% in the anti-H3 group or 5.8 ± 2.6% in the anti-H4 group (Fig. 1B).

Tissue levels of TNF-α and IL-6, two cytokines shown to be important in hepatic I/R,^21^ were also significantly decreased in the anti-histone antibody–treated I/R groups (Fig. 1C), with parallel results obtained from mouse serum (data not shown). Taken together, these results suggest that histones play a key role in the inflammatory response and organ injury following hepatic I/R.

### Extracellular Histones Exacerbate Hepatic I/R Injury

**Fig. 2:**
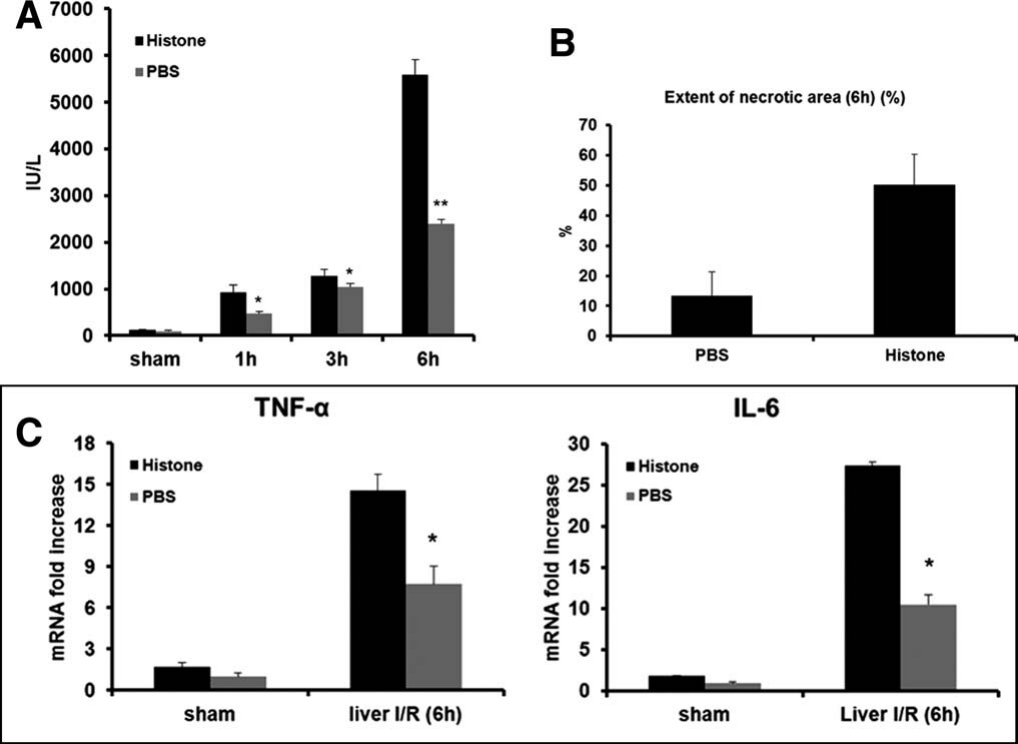
Treatment of exogenous histones mixture exacerbate liver I/R injury. (A) Sham-treated mice and mice that underwent ischemia and 1, 3, and 6 hours of reperfusion were treated with a nonlethal dose of exogenous histone mixture (25 mg/kg body weight) or vehicle PBS immediately after ischemia. sALT levels were analyzed. Data represent the mean ± SE (n = 12 mice per group). *P < 0.05. **P < 0.01. (B) Quantification of necrotic hepatocytes in hematoxylin and eosin–stained liver tissue. The graph is representative of liver sections from six mice per group. (C) Hepatic TNF-α and IL-6 mRNA. Data represent the mean ± SE (n = 6 mice per group). *P < 0.05.

To further explore the role of extracellular histones in I/R, exogenous histones were administered to mice. We found that a dose of 25 mg/kg did not cause elevation of sALT in sham-treated animals 7 hours after injection; consequently, this dose was used for all experiments. We also chose to administer histones immediately after ischemia, because we hypothesized that this approach was most physiologically relevant; additionally, we found that preinjection of histones 60 minutes prior to ischemia had little effect of sALT levels (Supporting Fig. 1). sALT levels measured 6 hours after reperfusion in mice that were given exogenous histones immediately after ischemia were significantly greater than in PBS-treated animals (Fig. 2A) (sALT 5,579 ± 340 IU/L versus 2,400 ± 87 IU/L). Liver histology also confirmed the hepatotoxic effect of exogenous histone administration. Histone-injected mice displayed 50.1 ± 10.2% necrotic hepatocytes versus PBS-treated mice at 13.4 ± 7% (Fig. 2B). Parallel tissue levels (Fig. 2C) and serum levels (data not shown) of TNF-α and IL-6 were observed. Because the dose of histones was nontoxic in sham-treated animals, but intensifying in I/R, these results suggest that administration of exogenous histones exacerbates organ injury only after I/R, serving to amplify the inflammatory cascade.

### Extracellular Histones Are Released from the Liver *In Vivo* After Hepatic I/R Injury and from Hepatocytes After Hypoxia *In Vitro*

**Fig. 3:**
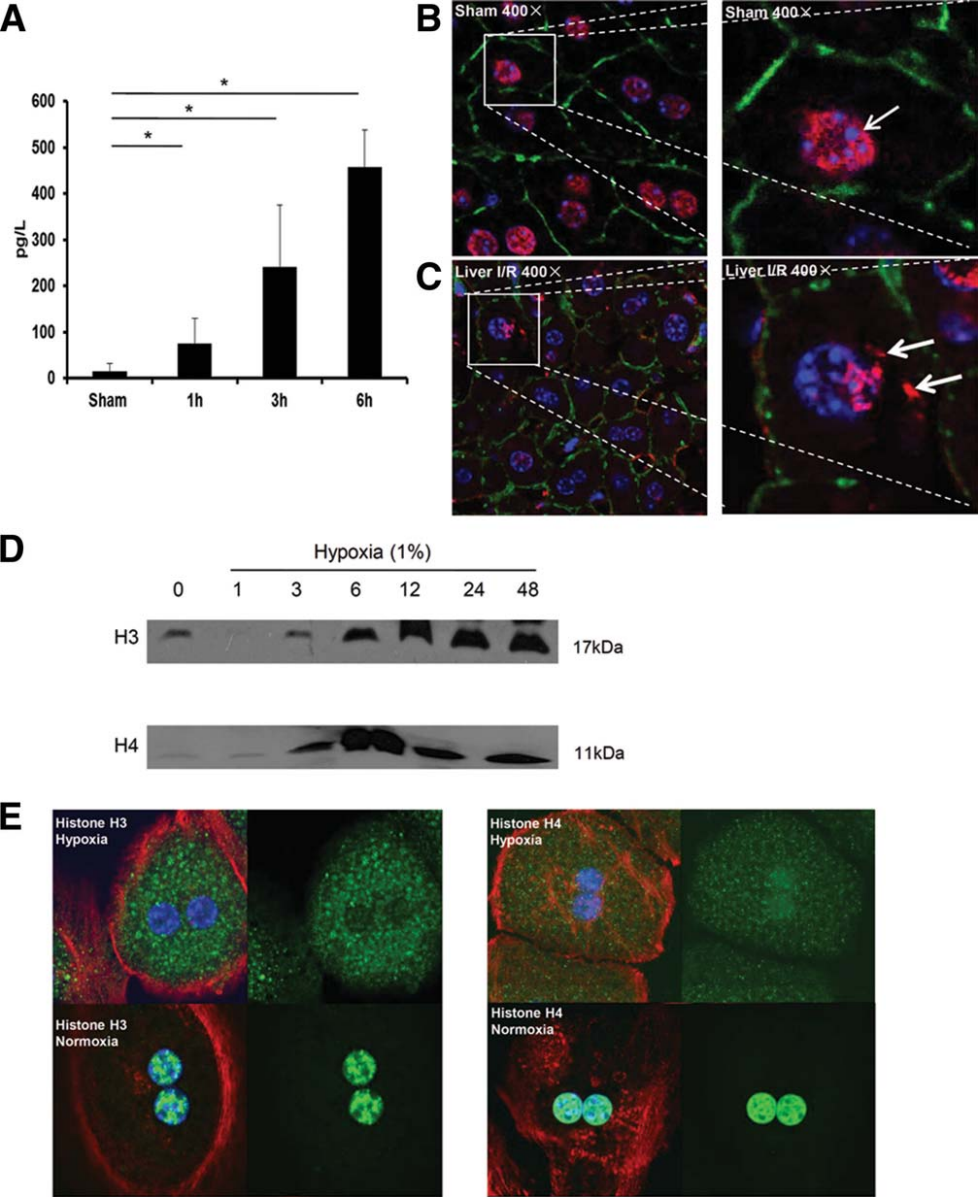
Extracellular histones are released from hepatocytes after hypoxia *in vitro* and from the liver *in vivo* after hepatic I/R. (A) Systemic histones levels were assessed by way of serum enzyme-linked immunosorbent assay. Data represent the mean ± SE (n = 6 mice per group). *P < 0.05. (B) Immunofluorescent stain of histone H3 from sections of normal liver and (C) and liver 6 hours I/R (original magnification ×400). Images are representative liver sections from six mice per group. Red, histone H3; blue, nuclei; green, F-actin. (D) Cultured mouse hepatocytes were exposed to hypoxia (1% O_2_) from 0 to 48 hours. Media were subjected to western blot analysis of histone H3 and H4. The blots shown are representative of three experiments with similar results. (E) Immunofluorescent stain of histone H3 and H4 from cultured mouse hepatocytes were exposed to hypoxia (1% O_2_) overnight (original magnification ×600). Images are representative of three experiments with similar results. Green, histone H3 or H4; blue, nuclei; red, F-actin.

To investigate whether histone-dependent injury was associated with extracellular changes in histone protein levels *in vivo*, enzyme-linked immunosorbent assay was performed on serum from animals subjected to liver I/R. After I/R, extracellular histone protein expression increased (Fig. 3A). We have shown hepatocytes to be the major source of the DAMP molecule HMGB1 after I/R,^23^ and necrotic hepatocytes are hypothesized to be a key source of endogenous DNA^24^; therefore, we explored hepatocytes as a potential source of endogenous extracellular histones. Immunofluorescent staining was performed in sham-treated livers and livers that underwent I/R. Histones localized to the nucleus of hepatocytes in sham-treated animals, and after I/R, histone-positive staining was observed in the cytoplasm of hepatocytes along with several areas that lacked nuclear histone staining (Fig 3Bb). We observed similar results in hepatocytes *in vitro* after hypoxia (Fig 3D,E), suggesting this translocation results in the heightened release of extracellular histones from hepatocytes to worsen I/R injury.

### Exogenous Histones Modulate Inflammatory Signaling Pathways

**Fig. 4:**
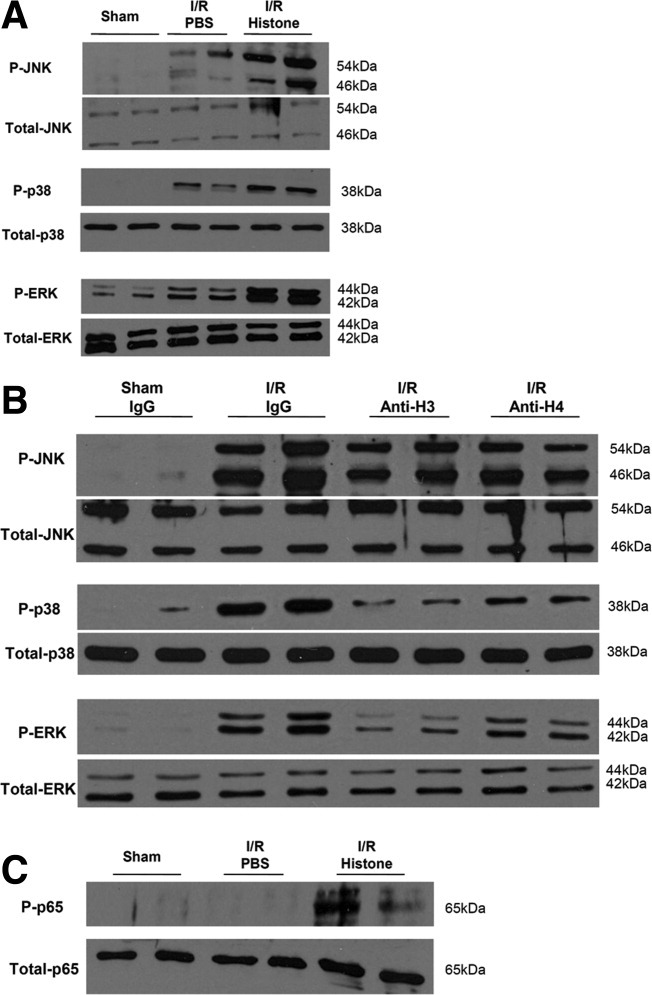
Extracellular histones modulate inflammatory signaling pathways. (A) Mitogen-activated protein kinase activation was determined in sham-treated mice and mice that underwent ischemia and 1 hour of reperfusion. Animals were treated with exogenous histones mixture or vehicle PBS. (B) Mitogen-activated protein kinase activation was determined. Hepatic protein lysates from ischemic lobes were obtained; each lane represents a separate animal. The blots shown are representative of three experiments with similar results. (C) Phosphorylation at serine 536 of the p65 subunit of NF-κB after 1 hour of I/R.

To determine how released extracellular histones might affect the inflammatory response to hepatic I/R injury, the role of histones in the activation of mitogen-activated protein kinases was evaluated. After 1 hour of I/R, phosphorylation of c-Jun N-terminal kinase, p38, and extracellular signal-regulated kinase increased, and these effects were further augmented with histone treatment (Fig 4A). Conversely, histone neutralization with anti-H4 or anti-H3 decreased phosphorylation of these proteins after I/R (Fig 4B). Finally, we observed an increase in NF-κB activation after 1 hour of I/R in liver tissue after histone treatment by phosphorylation at serine 536 of the p65 subunit (Fig. 4C).

### Extracellular Histones Mediate Hepatic I/R Injury Through TLR9

**Fig. 5:**
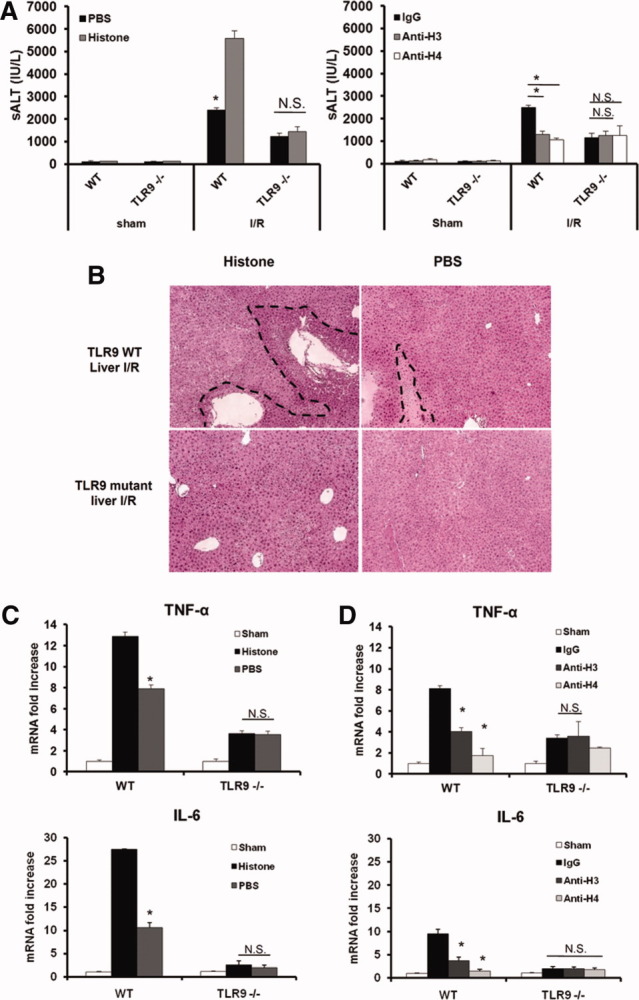
Extracellular histones mediate hepatic I/R injury through TLR9. (A) Serum ALT levels in TLR9 mutant and WT mice after I/R with either anti-histone antibodies or exogenous histone administration. Data represent the mean ± SE (n = 4-6 mice per group). *P < 0.05. (B) Hematoxylin and eosin–stained liver sections (original magnification ×100). Images are representative liver sections from six mice per group. The dashed line indicates the necrotic area. (C) Hepatic TNF-α and IL-6 mRNA in TLR9 mutant and WT mice after histone administration. (D) Hepatic TNF-α and IL-6 mRNA expression after histone neutralization. Results are expressed as the relative increase of mRNA expression compared with sham-treated animals. Data represent the mean ± SE (n = 4-6 mice per group). *P < 0.05. N.S., not significant.

Recent studies have demonstrated that TLR9-deficient mice are significantly protected from hepatic I/R injury,^9^ and our group has reported the role of TLR4.^8^ Thus, it is known that pattern recognition receptors play a critical role in sterile inflammation initiated by I/R injury. To determine whether the PRRs TLR9, TLR4, or TLR2 are involved in histone recognition during hepatic I/R injury, exogenous histones were administered to TLR9 mutant (TLR9^CpG/CpG^), TLR4 knockout (KO), TLR2 KO, or TRIF KO mice and their wild-type (WT; C57BL/6) counterparts. As expected, significant protection was observed in TLR9 mutant mice compared with their WT counterparts. However, exogenous histones failed to enhance liver damage in TLR9 mutant mice, whereas damage was significantly increased in TLR9 WT mice (Fig 5A). Whereas TLR9 mutant mice failed to respond to exogenous histones, we observed increased damage in TLR4 KO, TLR2 KO, and TRIF KO mice (Supporting Fig. 2). Additionally, administration of anti-H3 or anti-H4 to TLR9 mutant mice after I/R also failed to confer further protection, and examination of liver histology (Fig 5B) and tissue cytokine levels (Fig 5D) corroborated our sALT findings.

**Fig. 6:**
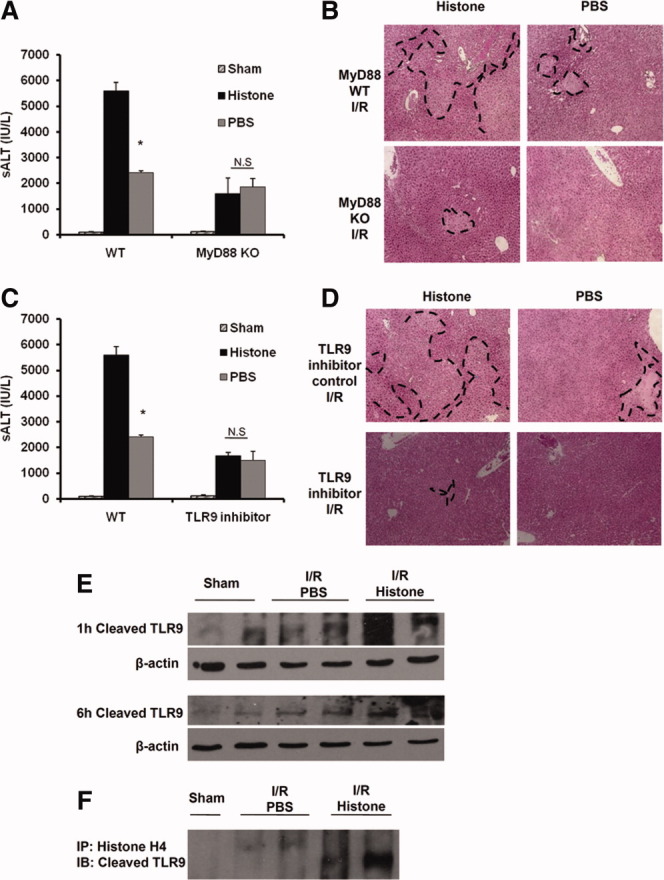
Extracellular histones-mediated hepatic I/R injury involves TLR9 signaling cascade. (A) Serum ALT levels in MyD88 KO and WT mice after 6 hours of I/R with or without histone administration. Data represent the mean ± SE (n = 4-6 mice per group). *P < 0.05. N.S., not significant. (B) Hematoxylin and eosin–stained liver sections (magnification ×100). Images are representative liver sections from six mice per group. The dashed line indicates the necrotic area. (C) sALT levels in ODN2088-treated mice after 6 hours of I/R. Data represent the mean ± SE (n = 4-6 mice per group). *P < 0.05. N.S., not significant. (D) Hematoxylin and eosin–stained liver sections from ODN2088-treated mice (magnification ×100). Images are representative liver sections from six mice per group. The dashed line indicates that necrotic area. (E) TLR9 activation by western blot analysis. The blots shown are representative of three experiments with similar results. (F) Coimmunopreciptation of histone H4 and cleaved TLR9 from liver cell lysated.

MyD88 is downstream of TLR9 signaling, hence MyD88 KO and WT mice were treated with exogenous histones after I/R. Extracellular histones also failed to enhance liver damage in MyD88 KO mice (Fig. 6A,B), revealing that histones function through MyD88–TLR9 signaling. To further confirm the role of TLR9 in histone recognition, we used the TLR9 antagonist ODN2088. Exogenous histones were administered to both ODN2088-treated and ODN2088 control–treated animals. Histones had no effect in TLR9 antagonist–treated WT mice (Fig. 6C). Liver histology was also consistent with these results (Fig. 6D).

The truncated form of TLR9, rather than the full-length form, functions to recruit MyD88 upon activation.^22^ Therefore, cleaved TLR9 was assessed after 1 hour and 6 hours of I/R. Cleaved TLR9 was enhanced compared with sham-treated mice and increased by way of histone treatment at both time points (Fig. 6E), demonstrating that histones further can enhance the activation of TLR9.

Finally, functional TLR9 was immunoprecipitated from whole liver tissue lysates in mice treated with PBS or exogenous histones. A physical interaction was detected between TLR9 and histone H4 in both PBS-treated and exogenous histone treated–mice after hepatic I/R, whereas no interaction was observed in sham-treated mice (Fig. 6F), further implicating the TLR9 signaling pathway in the mechanism of extracellular histone– mediated I/R injury. Taken together, these results suggest that TLR9 and its downstream signaling molecule MyD88 are involved in histone recognition during sterile inflammatory injury induced by I/R.

### Extracellular Histones Enhance Nucleic Acid–Mediated Inflammation Through TLR9

**Fig. 7:**
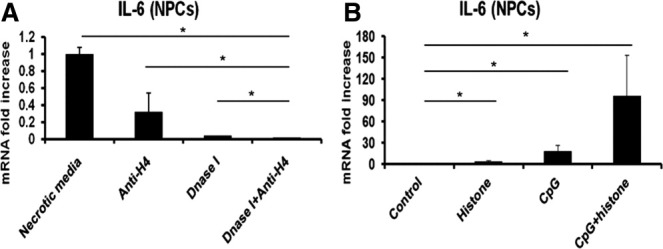
Extracellular histones enhances the nucleic acid–mediated damage after hepatic I/R. (A) IL-6 mRNA expression was obtained in NPCs cocultured overnight with media from necrotic hepatocytes. NPCs were treated with vehicle PBS, Dnase I, or anti-histone H4 antibody. Results are expressed as the relative increase of mRNA expression compared with PBS treatment. Data represent the mean ± SE and are representative of three experiments with similar results. *P < 0.05. (B) IL-6 mRNA expression was observed in NPCs that were treated with vehicle PBS, CpG, exogenous histones, or both. Results are expressed as relative increase of mRNA expression compared with PBS treatment. Data represent the mean ± SE and are representative of three experiments with similar results. *P < 0.05.

DNA from necrotic hepatocytes has recently been shown to increase hepatic NPC cytokine production in a TLR9-dependent manner.^9^ We used conditioned media from necrotic hepatocytes to stimulate hepatic NPCs. IL-6 mRNA significantly increased in NPCs after treatment with conditioned media, and this effect was reduced by treatment with DNAse. We observed a similarly significant effect when anti-H4 was added to the conditioned media, suggesting that histones also contribute to the stimulatory potential of necrotic cell supernatants (Fig 7A). Furthermore, the combination of DNAse and anti-H4 completely abolished IL-6 production. We also treated NPCs with low, nonstimulatory doses of CpG (ODN1826), histones, or both. With CpG or histones treatment alone, we observed minimal increase in proinflammatory cytokine IL-6 mRNA. However, cotreatment with CpG and histones led to a dramatic increase in IL-6 mRNA. Thus, we found a synergistic effect between nonstimulatory doses of CpG-DNA and exogenous histones (Fig 7B). Taken together, these results suggest that the inflammatory effects of histones may also occur by enhancing the DNA–TLR9 innate immune activation.

## Discussion

Nuclear histones are small, highly abundant proteins traditionally known as an essential component of nucleosomes in eukaryotic cells.^25^ Recently, Xu et al.^13^ showed that extracellular histones are involved in the pathogenesis of sepsis by contributing to endothelial cytotoxicity and triggering an inflammatory and thrombotic response *in vivo*. In addition, the cytotoxicity of histones appears to be regulated by proteolytic inactivation by activated protein C. Interestingly, activated protein C is protective in cardiac and liver I/R models,^26^, ^27^ and the proteolytic inactivation of histones represents a novel mechanism by which activated protein C protects against end organ damage.

TLR9 is an intracellular molecule that functions as a sensor of DNA, and it was originally reported that TLR9 KO mice failed to respond to bacterial DNA, which is rich in unmethylated CpG.^28^ Subsequently, TLR9 was shown to recognize endogenous DNA from mammalian cells.^29^ Recently, the critical role of TLR9 expressed on liver nonparenchymal cells was reported in the pathogenesis of liver I/R injury.^9^ DNA released from necrotic hepatocytes is thought to be the activating ligand of TLR9 signaling, although this has not yet been substantiated *in vivo*.

**Fig. 8:**
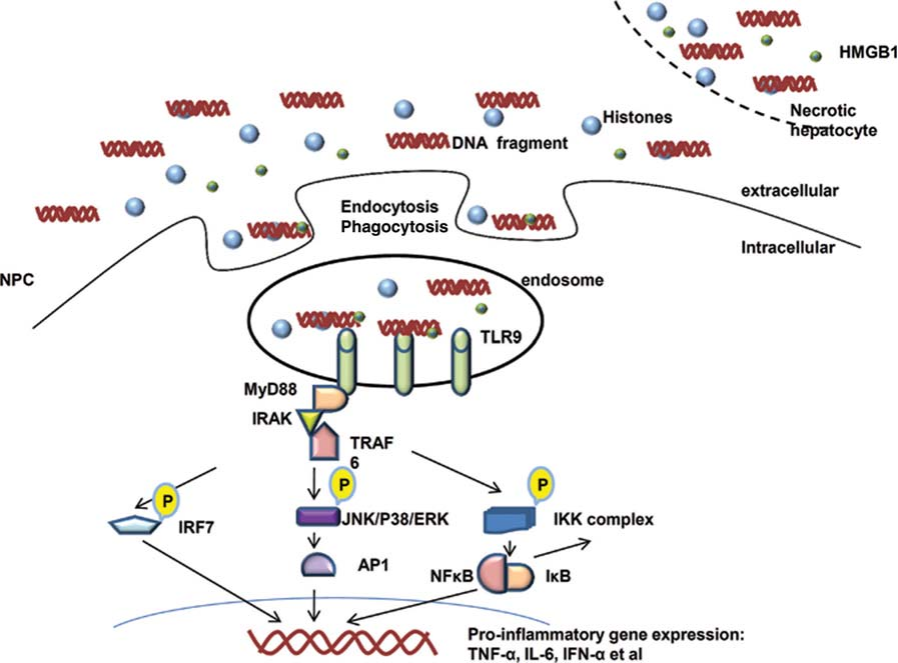
Hepatic I/R induces release of extracellular histones from hepatocytes. The model shows the release of extracellular histones after hepatic I/R.

Previously, we reported that HMGB and TLR4 are critical in I/R injury.^21^, ^23^ Here, we hypothesized that histones play a similar role as HMGB1 during liver I/R. We only observed hepatotoxic effects after I/R, suggesting that histones serve as cofactors to amplify other circulating pathogenic signals. This concept has been explored for other DAMPs, including HMGB1; HMGB1 combines with several endogenous and exogenous danger signals to amplify their effect.^30^ Thus, histones may also function to amplify the effect of circulating DNA through TLR9 activation. Regardless, the role of endogenous molecules released from necrotic and/or apoptotic cells remains uncertain. For acute, sterile inflammation induced by I/R, we hypothesize that the innate immune response is dependent on *in vivo* circulating complexes, including DNA, histones, HMGB1, and others (Fig. 8).

This study shows that endogenous serum histone levels increase significantly after I/R. However, whether extracellular histones are passively released from necrotic cells or actively secreted from damaged but viable cells is not yet known. Our group recently found that decreased nuclear histone deacetylase activity in hepatocytes after liver I/R contributed to the hyperacetylation and release of HMGB1.^20^ Further studies will determine whether posttranslational modifications of histones can contribute to extracellular histone-mediated toxicity.

In conclusion, the present study shows that extracellular histones similar to HMGB1 and DNA contribute to hepatic I/R injury by functioning as DAMPs, activating immune responses leading to inflammation and organ damage. The protective effects of blocking extracellular histones and the detrimental effects of exogenous histones in hepatic I/R are dependent on the activation of TLR9 signaling. Extracellular histones may also mediate sterile inflammation after hepatic I/R injury by enhancing the DNA-activated TLR9 signaling cascade. Thus, neutralization of extracellular histones may be a novel, effective strategy to minimize organ damage in the setting of ischemic liver injury.

## Abbreviations

DAMP: damage-associated molecular pattern
HMGB1: high-mobility group box 1
IgG: immunoglobulin G
IL-6: interleukin-6
I/R: ischemia/reperfusion
KO: knockout
mRNA: messenger RNA
NF-κB: nuclear factor κB
NPC: nonparenchymal cell
PAMP: pathogen-associated molecular pattern
PBS: phosphate-buffered saline
PCR: polymerase chain reaction
PRR: pattern recognition receptor
sALT: serum alanine aminotransferase
TLR: Toll-like receptor
TNF-α: tumor necrosis factor α
WT: wild-type
