# Supplementary figures and images for: Endogenous Histones Function as Alarmins in Sterile Inflammatory Liver Injury Through Toll-like Receptor 9 in Mice
Source: Hepatology. 2011 Aug 25;54(3):999–1008. doi: 10.1002/hep.24501 (PMC3213322; doi:10.1002/hep.24501)

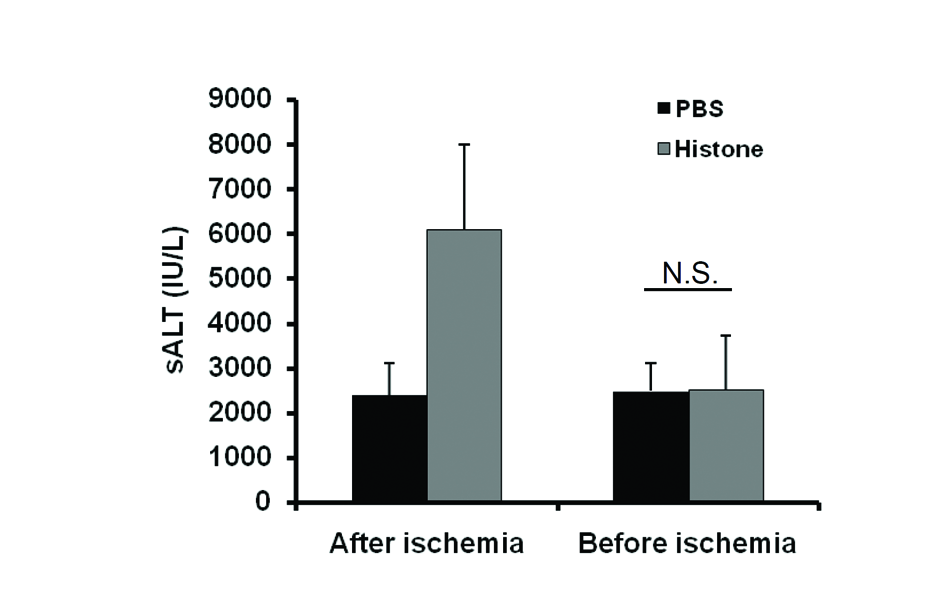

Supplement: Supplementary file 1 [file hep0054-0999-SD1.tif]

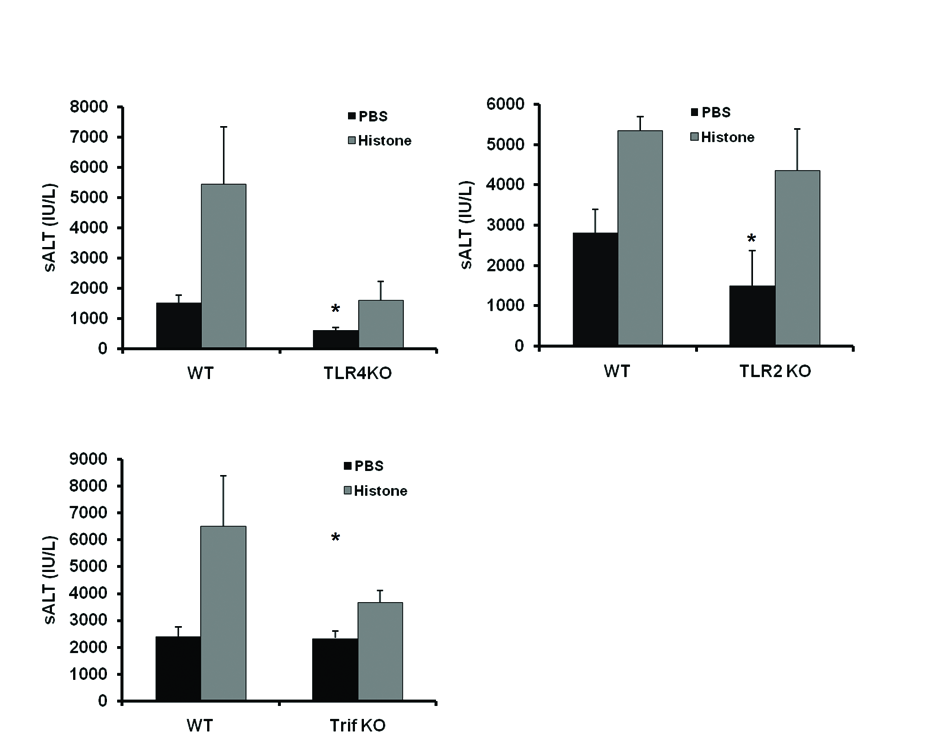

Supplement: Supplementary file 2 [file hep0054-0999-SD2.tif]
